# Analysis of initial laboratory diagnosis of malaria and its accuracy compared with re-testing from 2013 to 2018 in Yunnan Province, China

**DOI:** 10.1186/s12936-020-03477-1

**Published:** 2020-11-12

**Authors:** Ying Dong, Yan Deng, Yanchun Xu, Mengni Chen, Chun Wei, Canglin Zhang, Xianghua Mao, Jingbo Xue

**Affiliations:** 1 Yunnan Institute of Parasitic Diseases Control, Yunnan Provincial Key Laboratory, Yunnan Centre of Malaria Research, Pu’er, 665000 China; 2 National Institute of Parasitic Diseases, Chinese Center for Disease Control and Prevention, Shanghai, 200025 China

**Keywords:** Yunnan province, Malaria cases, Diagnosis, Re-testing, Accuracy

## Abstract

**Background:**

According to China’s Malaria Eradication Action Plan, malaria cases diagnosed and reported by health authorities at the county level must be further re-confirmed by provincial laboratories. The Yunnan Province Malaria Diagnostic Reference Laboratory (YPMDRL) began the synchronous implementation of microscopic examinations and nested polymerase chain reaction (nested-PCR) testing to re-test the malaria cases initially diagnosed by county-level laboratories and to evaluate the consistency of *Plasmodium* species identified between by YPMDRL and by the county-level laboratories from 2013 to 2018 in Yunnan Province.

**Methods:**

Data on malaria initial diagnosis completed by county-level laboratories in Yunnan Province were collected weekly from the “China Disease Prevention and Control Information System” from 2013 to 2018. The YPMDRL performed *Plasmodium* microscopic examination and 18S rRNA gene nested-PCR testing on every malaria case managed by the China Disease Prevention and Control Information System. The re-testing detection results were fed back to the initial diagnosis and reporting unit for revision of malaria case types.

**Results:**

A total of 2,869 malaria cases were diagnosed and reported by county-level laboratories in Yunnan Province from 2013 to 2018. The re-testing rate was 95.6% (2,742/2,869), and the re-testing rate increased from 2013 to 2018. Among the re-tested 2,742 cases, 96.7% (2651/2742), 2.2% (59/2742), and 1.1% (32/2742) were doubly examined by microscopy and by nested-PCR, only by microscopy, and only by nested-PCR, respectively. The total *Plasmodium* species accuracy rate at county-level laboratories was 92.6% (2,543/2,742) reference to the diagnosis by YPMDRL. Among the inconsistent 199 cases, they were identified as including 103 negative cases, 45 falciparum malaria cases, 30 vivax malaria cases, 11 ovale malaria cases, and 10 malariae malaria cases by YPMDRL. From 2013 to 2018, the revised and registered malaria cases by the China Disease Prevention and Control Information System in Yunnan Province was 2,747 cases, including 2,305 vivax malaria cases*,* 421 falciparum malaria cases, 11 ovale malaria cases, and 10 malariae malaria cases.

**Conclusions:**

The double re-testing strategy by microscopy and by gene testing increases the accuracy of diagnoses malaria in Yunnan Province, and gene testing can reliably differentiate *Plasmodium* species. The re-testing results provided by YPMDRL are the authoritative basis for revising malaria kind in Yunnan Province.

## Background

The People’s Republic of China enacted a new law on the prevention and control of infectious diseases in 2004 [1]. Under the terms of this law, malaria is categorized as a B Class infectious disease, and every administrative county in China is responsible for the prevention and control of malaria and for reporting malaria epidemic information. In addition, epidemic information reporting should be procedural and standardized with patterns and time limits. The China Information System Disease Control and Prevention (CISDCP) addressed the need for efficient collection and transmission of epidemic information on infectious diseases in China from county-level to prefecture-level, province-level, and national-level institutions of disease prevention and control [2]. The 24-h requirement to report the initial diagnosis of a malaria case in CISDCP is especially beneficial in advancing the timely collection of data to accurately analyse the malaria epidemic situation in China [3–5]. The number of malaria cases in China has been reported and published from CISDCP system in the past decade [6–8].

However, effective collection and analysis of malaria epidemic data cannot be separated from the accurate diagnosis of each malaria case [9]. External quality evaluations [10] or establishment of regional reference laboratories [11] are two practical methods to improve the quality of malaria laboratory diagnoses. Ethiopia conducted a month-long tracking and evaluation of *Plasmodium* microscopy skills on 60 laboratory personnel in 2015, and only 8.3% (5/60) of the participants reached the professional level required to correctly identify *Plasmodium* species [9], and the agreement rate of *Plasmodium* infection was only 71.2% (198/276) [12]. However, the accuracy rate of species identification was significantly improved after personnel received training in *Plasmodium* microscopy skills (OR = 7.0) [12]. As a non-malaria-endemic country with an average annual diagnosis of 1500 imported malaria cases [13–17], the United States not only pay attention to timely reporting of malaria diagnosis results, but also ensures diagnostic accuracy and proper identification of *Plasmodium* species through cross-checking between laboratories [18]. Only 79.1% (706/893) of the 893 malaria cases initially diagnosed in Belgium were confirmed to be the correct species by microscopic diagnosis in the national-level reference laboratory between 2013 and 2017 [11]. Therefore, the World Health Organization (WHO) advocates the use of cross-examination in reference laboratories to improve the diagnostic quality of laboratories in malaria endemic areas [19].

The Yunnan Province Malaria Diagnostic Reference Laboratory (YPMDRL) was formally certified as a member of the China Malaria Diagnosis Reference Laboratory Network in 2012 [20], and began to assume the responsibility of re-testing of malaria cases initially diagnosed [21] and assessment of negative samples from county-level laboratories in Yunnan Province, which it was confirmed that there was no false negative diagnosis from 2016 to 2018 (Additional file 1). It has been operating normally for nearly eight years and has re-tested the most malaria cases as well as the last three indigenous malaria cases during the stage of Elimination Malaria Programme (EMP) in China [6–8]. The re-testing results of previous years have also been used to revise the *Plasmodium* species initially diagnosed by the county-level laboratories in Yunnan Province. In order to introduce the re-testing work of YPMDRL to the peer and explore ways to ensure the continuous stability and improvement of laboratory diagnostic capacities in Yunnan Province after EMP, this paper conducted this systematic analysis to verify the accuracy of laboratory diagnostics against re-testing in Yunnan Province from 2013 to 2018.

## Methods

### Ethics statement

The study was approved by the Yunnan Institute of Parasitic Diseases and Ethical Committee. Genetic testing was performed on stored blood samples obtained as part of the routine diagnostic work-up of patients with fever who were suspected to have malaria. Although there was no risk and the data processing after sample collection was done anonymously, informed consent was obtained.

### Data collection of initial malaria diagnoses

Data was retrieved from the CISDCP database through the infectious disease report management module. From 2013 to 2018, the “report place” and “entry time” as search terms were used to search for cases of malaria disease on Monday of each week. Exported data included the case ID number, diagnosis time, disease type, initial diagnosis institution, and case report place. According to the epidemiological investigation, the sources of malaria infection were determined as follows: indigenous infection cases included those who had no history of travel to epidemic areas outside Yunnan Province within 30 days before the onset of malaria; imported infection cases included those who had a history of migration from malaria endemic areas, such as Myanmar and Africa, within 30 days of malaria onset.

### Blood sample collection and transport

Two peripheral blood samples from every malaria patient with a clinical attack were collected by county-level malaria diagnostic laboratories in their administrative jurisdiction. The blood samples were used for microscopic examination in the county-level laboratories to initially diagnose for malaria cases, and were used to prepare another’s blood smears and dried blood spot samples for re-testing in YPMDRL. The latter were sent twice each month to YPMDRL by postal service. When there are few cases, the blood samples may be sent at any time to YPMDRL.

### YPMDRL re-testing malaria cases samples

Blood smears from the initially diagnosed malaria cases were examined by microscopy at the YPMDRL according to the methods of Dong et al. [21] and Zhu et al*.* [22], and others [23], and the four species of human *Plasmodium* were identified according to their morphological differences, as seen in thin blood smears. According to the methods of the literature [21, 23–26] and Laboratory Diagnosis Programme of EMP, nested polymerase chain reaction (PCR) was performed on each case simultaneously to amplify the unique region of 18S ribosomal RNA (rRNA) gene of *Plasmodium falciparum*, *Plasmodium vivax*, *Plasmodium malariae* and *Plasmodium ovale* and to distinguish whether the *Plasmodium* infection was single or mixed. Primer details of nested PCR and their reaction cycle were showed in Additional file 2.

The *Plasmodium* species was judged according to detection at the YPMDRL as follows: (1) the case was determined as negative when the microscopic examination and PCR testing were both negative for *Plasmodium* infection; (2) when *Plasmodium* infection was only detected by either microscopic examination or PCR testing, the final species was decided by the result of positive method; (3) PCR testing was used to determine the *Plasmodium* species when there was a difference between the microscopic examination and the PCR testing results. The re-testing examinations were performed at the YPMDRL, and determination results were provided once a month to the county-level laboratories to revise their initial diagnoses.

### Primary index and statistical analysis

The database and information, which included the type of malaria, location of initial diagnosis and reporting, source of malaria infection, and re-testing results by the YPMDRL. SPSS 16.0 software (SPSS, Inc., Chicago) was used to analyse the number of malaria cases initially diagnosed, number of re-tested diagnoses, and so on. Among these analyses, the number of re-tested diagnoses (PTD) was counted, which may be re-tested by both microscopic examination and by PCR testing at same time [21, 23–26] or only re-tested by one of the two detection methods. Test species accuracy (TSA) refers to the consistency between the *Plasmodium* species identified by YPMDRL and the species initially diagnosed by county-level laboratories, for assessment the accuracy of the initial diagnosis. The Chi-square test was performed to test the correlation on the re-testing rate or the consistency rates of two laboratories (i.e. the accuracy rate of the initial diagnosis) in different years. The significance level was 0.05, and ArcGIS 10.1 (Environmental Systems Research Institute, ESRI) was used to map the malaria cases.

## Results

### Malaria cases initially diagnosed

**Fig. 1:**
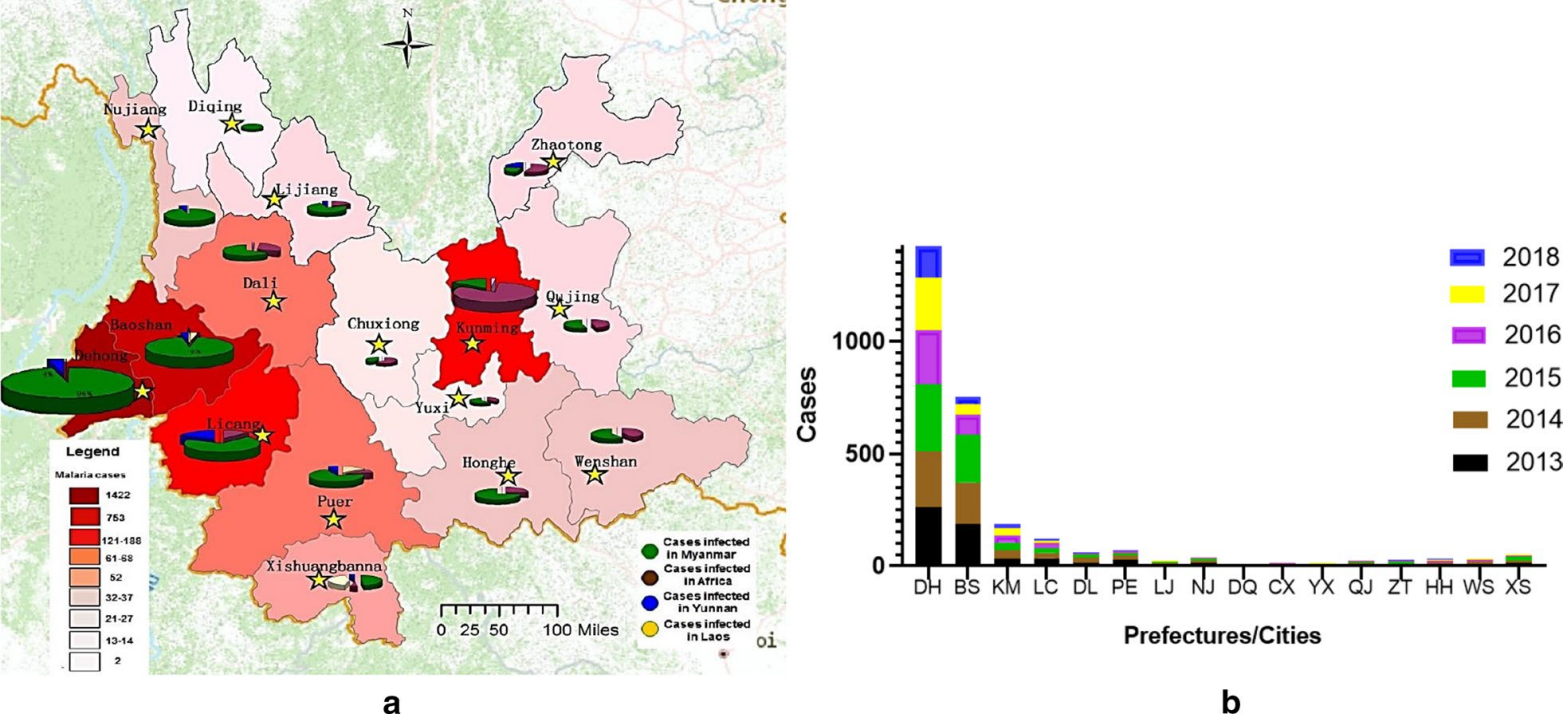
Region (**a**) and time (**b**) distribution of initial diagnosed malaria cases in Yunnan Province from 2013 to 2018. (1) **a**: Red from dark to light indicates the number of malaria cases found during study period from more to less, including Dehong(DH), Baoshan (BS), Kuming (KM), Lijiang (LJ), Pu’er (PE), Dali (DL), Xishuangbanna (XS), Nujiang (NJ), Weishan (WS), Honghe (HH), Zhaotong(ZT), Qujing (QJ), Licang (LC), Yuxi (YX), Chuxiong(CX), and Diqing(DQ) 16 prefectures in Yunnan Province.(2) **b**: The time distribution of malaria cases coming from every prefectures

There were 2869 malaria cases initially reported by county-level laboratories in Yunnan Province, including 2355 cases of *Plasmodium vivax,* 453 cases of *Plasmodium falciparum,* and 61 cases without *Plasmodium* species classification from January 2013 to December 2018. Among them, 2,747 cases were diagnosed by microscopic examination, and the others 122 cases were clinically diagnosed. The number of infected malaria cases from Africa, Myanmar, Laos, Cambodia, and Yunnan was 397, 2380, 18, and 74, respectively. Initially diagnosed and reported cases covered 16 prefectures in Yunnan Province, including 1422 cases in Dehong, 753 cases in Baoshan, 188 cases in Kunming, 121 cases in Lincang, 68 cases in Pu’er, 61 cases in Dali, 24 cases in Qujing, 34 cases in Wenshan, 27 cases in Zhaotong, 52 cases in Xishuangbanna, 21 cases in Lijiang, 2 cases in Diqing, 37 cases in Nujiang, 14 cases in Yuxi, 13 cases in Chuxiong, and 32 cases in Honghe (Fig. 1a). From 2013 to 2018, the number of malaria cases diagnosed and reported by every prefecture (or city) was shown in Fig. 1b, and Dehong prefecture initially diagnosed and reported the most malaria cases.

### Analysis of YPMDRL re-testing results

Among the 2,869 malaria cases initially diagnosed, 2742 cases were re-tested by the YPMDRL, with a re-testing rate of 95.6% (2742/2869). The re-testing rates increased annually from 2013 to 2018, and the differences was statistically significant (*χ*^2^ = 19.925, *P* < 0.05) (Table 1). Of the cases initially diagnosed, 127 (4.4%) (127/2,869) were not re-tested. Among not re-tested cases, 19 cases were excluded from *Plasmodium* infection after 24 h owing to be confirmed as other febrile diseases, and 108 cases could not be re-tested due to lack of blood samples. These non-re-tested cases numbered 29 (26.8%) cases in 2013, 22 (20.4%) cases in 2014, 34 (31.5%) cases in 2015, 14 (13.0%) cases in 2016, 7 (6.5%) cases in 2017, and 2 (1.9%) cases in 2018, It showed that the proportion of missing re-testing in YPMDRL was decreasing year by year (*χ2* = 13.325, *P* < 0.05).

**Table 1:** Analysis of malaria re-testing diagnosis in Yunnan Province from 2013 to 2018

Years	No. ID	No. PTD (PTDR)	Re-testing ‘s method			Consistency of MIC & PCR re-testing		Re-testing by MIC		Re-testing by PCR	
			MIC &	Only	Only	No. MIC & PCR	No. CMPD (CMPDR)	No. MIC	No. MSA (MSAR)	No. PCR	No. RSA (RSAR)
			PCR	MIC	PCR						
			(CR)	(CR)	(CR)						
2013	624	588	550	22	16	550	517	572	512	566	507
		(94.2)	(93.5)	(3.8)	(2.7)		(94.0)		(89.5)		(89.6)
2014	584	556	542	12	2	542	510	554	508	544	495
		(95.2)	(97.5)	(2.2)	(0.3)		(94.1)		(91.7)		(91.0)
2015	659	619	591	15	13	591	578	606	564	604	553
		(93.9)	(95.5)	(2.4)	(2.1)		(97.8)		(93.1)		(91.6)
2016	428	414	408	5	1	408	391	413	382	409	376
		(96.7)	(98.6)	(1.2)	(0.2)		(95.8)		(92.5)		(91.9)
2017	346	339	335	4	0	335	333	339	310	335	311
		(98.0)	(98.8)	(1.2)			(99.4)		(91.4)		(92.8)
2018	228	226	225	1	0	225	225	226	209	225	207
		(99.1)	(99.6)	(0.4)			(100.0)		(92.5)		(92.0)
Total	2869	2742	2651	59	32	2651	2554	2710	2485	2683	2449
		(95.6)	(96.7)	(2.2)	(1.1)		(96.3)		(91.7)		(91.3)
*χ*^*2*^, *P*	,	19.925 0.001					37.625 < 0.0001		5.646 0.342		3.562, 0.614

*ID* Initial diagnosis, undertaken by county laboratory, *CR* Constituent ratio, *PTD* re-testing diagnosis, undertaken by YPMDRL, *MIC* Microscopy test, *CMPD* The Consistency of MIC & PCR re-testing by YPMDRL for assessment the stability of two re-testing tests, *CMPDR* The rate of CMPD; *MSA* Microscopy test species accuracy comparison ID with PTD for assessment the accuracy of microscopic examination in county-level laboratories, *MSAR *Microscopy test species accuracy rate, *RSA* PCR test species accuracy comparison ID with PTD for assessment the accuracy of microscopic examination in county-level laboratories; *RSAR* PCR test species accuracy rate

Among the re-tested 2742 malaria cases, the constituent ratio of cases re-tested by a combination of microscopy and PCR testing, only by microscopy, and only by PCR testing were 96.7% (2651/2742), 2.2% (59/2742), and 1.1% (32/2742), respectively. It showed that most malaria cases were double re-tested by microscopy and by PCR testing in YPMDRL.

Of the re-tested malaria cases, the *Plasmodium* species of 2485 cases identified by microscopy examination in YPMDRL were consistent with initial diagnosis, with a microscopy species accuracy rate (MSAR) of 91.7% (2485/2,710). From 2013 to 2018, the differences between the MSARs were not statistically significant (*χ*^*2*^ = 5.646, *P* > 0.05) (Table 1). The *Plasmodium* species of 2,449 malaria cases identified by PCR testing in YPMDRL were consistent with initial diagnosis, with a PCR species accuracy rate (RSAR) of 91.3% (2449/2683). From 2013 to 2018, the differences between the RSARs were not statistically significant *(χ*^*2*^  = 3.562, *P* > 0.05) (Table 1). It indicated that the improvement of microscopic technology at county-level laboratories were not obvious.

On the other hand, the consistency between microscopic examination and PCR testing by YPMDRL had been high and increasing from 2013 to 2018,the differences between the CMPCRs were statistically significant (*χ*^*2*^ = 37.625, *P* < 0.05) (Table 1), which showed that the technology of microscopic examination and PCR detection undertaken by YPMDRL were trustworthy.

Although the re-testing rate gradually increased from 2013 to 2018 in Yunnan Province (Table 1). The prefectures that diagnosed more malaria cases, such as Dehong and Baoshan (Fig. 1b), had higher accuracy rate of *Plasmodium* species identification. The *Plasmodium* species accuracy rate in Dehong ranged from 94.8% (221/233, 2014) to 97.8% (135/138, 2018), which was higher than the average of the province as a whole (92.8%, 516/556 and 92.9%, 210/226) and the differences between the TSARs were statistically significant (*χ*^*2*^ = 23.175, *P* < 0.05) (Additional file 3). In addition, from 2013 to 2018, there were more and more prefectures achieving 100% species accuracy in Yunnan Province. Especially after 2016, the number of these prefectures reached to more than 7, accounting for 43.8% (7/16) (Additional file 3).

Among the re-tested 2742 cases, there were199 cases to be proved as the inconsistent between initial diagnoses and the re-testing diagnosis, accounting for 7.3% (199/2742). Of the 199 cases, 75 cases originally diagnosed as *P. falciparum* infection were re-tested as *Plasmodium* negative in 44 cases, *P. vivax* infection in 30 cases, and *P. ovale* infection in 1 case. The other 124 cases originally diagnosed as *P. vivax* infection were re-tested as *Plasmodium* negative in 59 cases, *P. falciparum* infection in 45 cases, *P. ovale* infection in 10 cases, and *P. malariae* infection in 10 cases. Total species incorrect samples were 96 (Additional file 4). The total incorrect percentage of the initial diagnoses including *P. falciparum*, *P. ovale*, and *P. malariae* species was 52.4% (65/124), which was higher than the incorrect proportion of only counting *P. vivax* species (41.3%, 31/75). Although the number of *Plasmodium* species incorrectly identified gradually decreased from 2013 to 2018, the differences between NARs (The rate of species incorrect of initial diagnosis comparison with re-testing diagnosis by YPMDRL) was no statistically significant (*χ*^*2*^ = 0.191, *P* < 0.05) ( Additional file 4).

### Revise the initial diagnosis

**Fig. 2:**
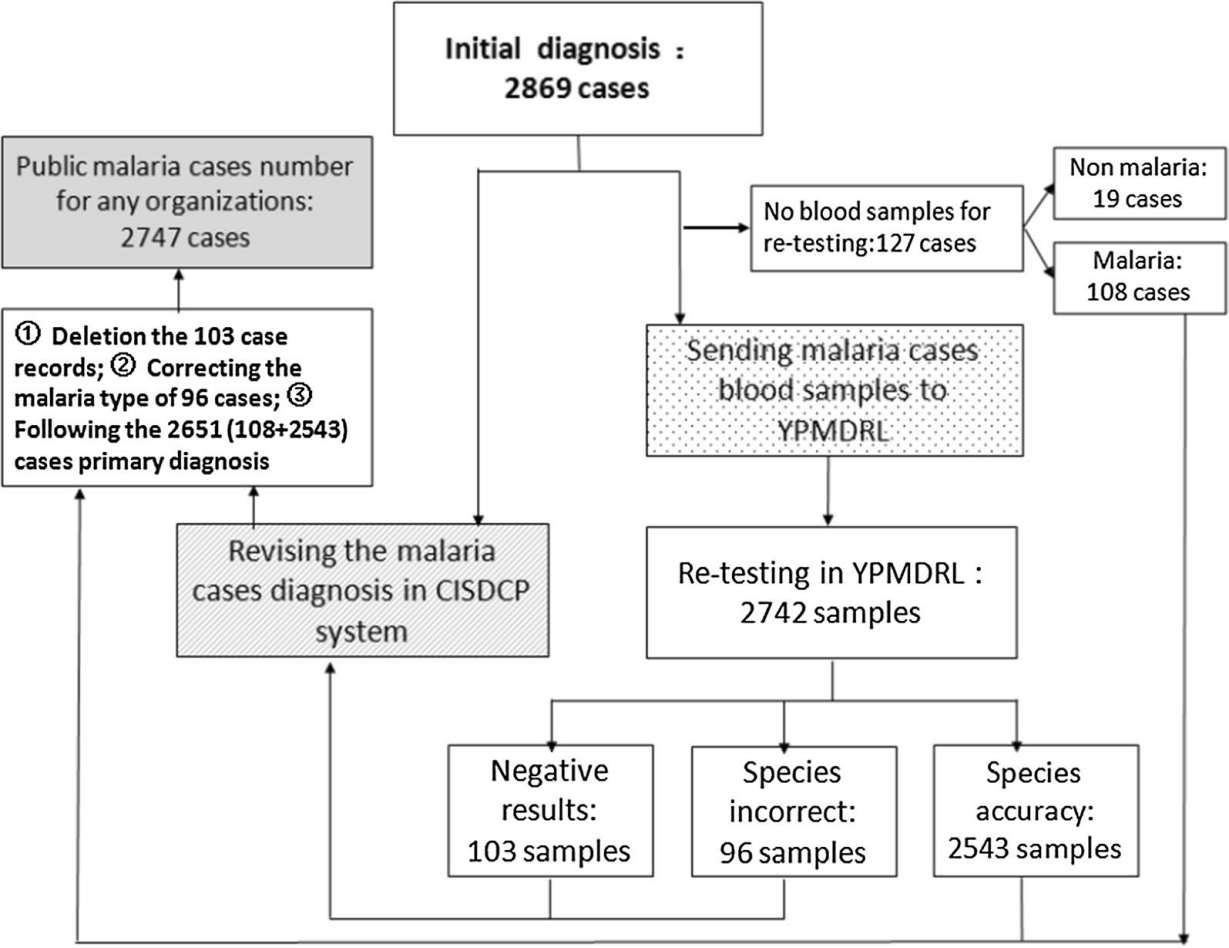
Management and revision flow chart of the re-testing results from YPMDR

From 2013 to 2018, 19 of 2869 malaria cases initially diagnosed were deleted in the CISDCP system due patients being diagnosed as other febrile diseases within 24 h. Another 103 cases were also deleted because they were *Plasmodium* negative after re-testing. The *Plasmodium* species of 96 cases were corrected according to the re-testing results from YPMDRL. Of the 2869 cases, 2543 retained the initial diagnosis because the *Plasmodium* species was correctly identified at county-level laboratories, and 108 missing re-testing samples also retained the initial diagnosis because they lacked the re-testing results. Finally, a total of 2747 malaria cases were final disclosure (Fig. 2). Among these cases, there were 2305 cases of *P. vivax*, 421 cases of *P. falciparum*, 11 cases of *P. ovale,* and 10 cases of *P. malariae*.

## Discussion

The China Malaria Diagnosis Reference Laboratory Network was established in 2012 and aimed to regulate China's provincial-level laboratories to re-testing examination of the malaria cases initially diagnosed by county-level laboratories. The recommended re-testing methods for *Plasmodium* infection included microscopic examination and/or genetic testing [24]. The genetic testing method was used to differentiate *Plasmodium* species when microscopy could not confirm various species. Snounou’s nested-PCR method [24], with high specificity and sensitivity was usually adopted to detect *Plasmodium* infection [27]. On account of the fact that the nested PCR amplification products could not only be directly used for DNA sequencing and analysing of the gene polymorphism of *Plasmodium*, Yunnan Province still applied the nested PCR based on Snounou's method to re-test *Plasmodium* infection during EMP, even though the real-time fluorescence quantitative PCR was faster [28, 29] and more sensitive [30] in genetic diagnosis of malaria. In order to better control the internal quality evaluation, the YPMDRL persists in the strategy that every malaria case originally diagnosed must be simultaneously re-tested by microscopic examination and by gene testing [21, 24]. By the end of 2014, the consistency of the two methods conducted by the YPMDRL reached 97.4% [21]. The 95.6% (2742/2,869) re-testing rate for malaria cases initially diagnosed in Yunnan province in nearly six years was significantly higher than 45.5% from the WHO report 2018 [8].

Moreover, the practice of identifying *Plasmodium* species based on gene testing could also help microscopists in Yunnan Province to correct their understanding of the unfamiliar morphology of *Plasmodium*. In recent years, *P. falciparum* infection cases imported from Africa have increased in Yunnan Province [31]. Once these falciparum malaria cases imported from Africa cause a clinical outbreak, the *Plasmodium* morphology in peripheral blood often appears as mature trophozoites. This morphology of *P. falciparum* was easily confused with the immature trophozoites of *P. vivax* before 2016 in Yunnan Province [21, 32], with the error rate of 55.6% in 2016 (Additional file 3). Moreover, a lack of familiarity with the morphology of *P. ovale* and *P. malariae* is also another major cause leading to incorrect identification of *Plasmodium* various species in county-level laboratories in Yunnan Province (Additional file 3).

Since 2013, 20 cases of *P. ovale* infected from Africa [27] and *P. malariae* infected from Myanmar were identified during the re-testing at the YPMDRL [31], but all these cases were misdiagnosed as *P. vivax* in county-level laboratories. It is possible that microscopists ignored to observe the morphological changes of erythrocytes parasitized by non-falciparum *Plasmodium* species. It was observed [33–36] that the different growth stages of *Plasmodium,* such as trophozoites, schizonts and gametocytes with different proportions, appeared simultaneously in the peripheral blood of patients with *P. vivax*, *P. malariae* and *P. ovale*. However, the morphology changes of erythrocytes were significantly different between three non-falciparum *Plasmodium* species, which the erythrocytes parasitized by *P. vivax* were generally enlarged, those parasitized by *P. malariae* were generally smaller than normal ones, and those parasitized by *P. ovale* were oval in shape [32] and contained obvious Schüffner's dots [37].

Nevertheless, the species accuracy rate of malaria initially diagnosed in Yunnan Province remained above 92% (Additional file 2) from 2013 to 2018, which is significantly higher than the 62–66% accuracy rate that Yin et al. reported in China in 2015 [20]. A potential reason for this could be the different microscopists selected for assessment between the two studies. In the Yin et al*.* study, those personnel were not always specialized malaria microscopists, but testers who could undertake the pathogenic testing for multiple parasitic diseases at the same time except for *Plasmodium*. Retention of multiple testing skills may have limited microscopists in mastering *Plasmodium* identification. Moreover, there were significant regional differences in the species accuracy rate between prefectures in Yunnan Province. Generally, in those prefectures that are traditional malaria endemic areas with a high number of diagnosed malaria cases, such as Dehong and Baoshan, the species accuracy rate was higher than the prefectures that are non-traditional malaria endemic areas with a low number of diagnosed malaria cases, such as Kunming and Zhaotong. The phenomenon that laboratories in areas with more diagnosed malaria cases have a stronger ability to precisely identify *Plasmodium* species is consistent with the findings of studies conducted by Abanyie et al. [18], Loomans et al. [11], Alemu et al*.* [12], and Mukadi et al*.* [38]. It is suggested that continuous practice is advantageous in maintaining the microscopic skills required to examine *Plasmodium* at a high level and stable state. In the next stage, it is necessary to strengthen the training of microscopic examination skills for *P. malariae* and *P. ovale*, and also how to distinguish between *P. ovale curtisi* and *P.ovale wallikeri* on the basis of gene polymorphism information analysis.

*Plasmodium* density was considered an effective indicator in predicting the severity of a malaria attack [10, 39], but the China Malaria Diagnostic Criteria does not require *Plasmodium* density to be measured during malaria diagnosis [22]. Therefore, the *Plasmodium* density was not collected during the initial diagnosis of malaria cases in Yunnan Province from 2013 to 2018, and the *Plasmodium* density data was not also analysed in this paper. Additionally, although the organizer of EMP required YPMDRL to collect the every blood samples of malaria cases initial diagnosed and re-test their *Plasmodium* infection at the same time, there were still some accidents, such as patients’ refusing to supply blood or too litter blood samples not enough to prepare the blood smears and dried blood spot specimens for re-testing, etc., that resulted in the missing (3.8%, 108/2869) or delayed collection (3.8%, 103/2742) for re-testing specimens. However, considering that the elimination malaria and consolidation its effect were a long-lasting monitoring plan, the few shortcomings in the programme design should be improved in the future. In addition, the introduced working model of implementation reference laboratory re-testing and application its’ results to revise the identification of malaria species in Yunnan Province are worthy of promotion in the other malaria elimination implementation areas.

## Conclusions

The proportion of the malaria cases re-tested by the YPMDRL has been higher than 93% since 2014. The species accuracy of malaria initially diagnosed by microscopy in county-level laboratories has been higher than 92% from 2013 to 2018. This finding partially clarified the improper understanding that the level of correct identification of *Plasmodium* species is not high in the basic level laboratories in Yunnan Province. The strategy of double re-testing both by microscopy and by gene testing ensures to diagnose malaria cases more precisely in Yunnan Province. The re-testing results from YPMDRL are the main basis to revise the malaria kinds during EMP in Yunnan Province.

## Supplementary information

**Additional file 1.** Re-testing of negative samples by YPMDRL in Yunnan Province (2016 to 2018).

**Additional file 2.** The details of nested PCR testing for differentiating between various *Plasmodium* species.

**Additional file 3.** Accuracy of initial malaria diagnoses in Yunnan Province prefectures (2013 to 2018).

**Additional file 4.** Analysis of the inconsistency between initial diagnosis and re-testing in Yunnan Province from 2013 to 2018.

## Data Availability

Not applicable.

## Abbreviations

CISDCP: China information system disease control prevention
YPMDRL: Yunnan province malaria diagnostic reference laboratory
EMP: Elimination malaria programme
ID: Initial diagnosis
MIC: Microscope
MSA: Microscope species accuracy
MSAR: Microscope species accuracy rate
PTD: Re-testing diagnosis
PTDR: Re-testing diagnosis rate
RSA: PCR test species accuracy
RSAR: PCR test species accuracy rate
SA: Species accuracy
CMPD: The Consistency of MIC & PCR re-testing by YPMDRL
CMPDR: The rate of CMPD
