# Supplementary material for: Analysis of initial laboratory diagnosis of malaria and its accuracy compared with re-testing from 2013 to 2018 in Yunnan Province, China
Source: Malar J. 2020 Nov 12;19:409. doi: 10.1186/s12936-020-03477-1 (PMC7664069; doi:10.1186/s12936-020-03477-1)
Supplement: Supplementary file 1 — Additional file 1. Re-testing of negative samples by YPMDRL in Yunnan Province (2016 to 2018). [file 12936_2020_3477_MOESM1_ESM.docx]

**Additional file 1**

Re-testing of negative samples by YPMDRL in Yunnan Province (2016 to 2018)

**Method：**

As a monitoring measure to find the malaria infection source in time, 1% of the population in Yunnan Province needs to be tested for *Plasmodium* infection every year. The monitored objects usually were "four kinds of fever patients", that is, fever patients with initial diagnosis for malaria, fever patients for suspected malaria, fever patients for suspected influenza and fever patients with unknown etiology. The light microscopy examination was used to find *Plasmodium* infection. The monitoring measure was completed by the county-level malaria diagnosis laboratory.

When the monitored objects were found to infect *Plasmodium* by microscope examination in county-level laboratory, these objects were initially diagnosed as malaria cases, their blood samples would be sent to the Yunnan Province Malaria Diagnostic Reference Laboratory (YPMDRL) for re-testing again both by microscopic examination and by genetic testing. For the negative blood samples without *Plasmodium* infection, they were selected by using stratified random sampling method for re-testing in prefecture-level laboratories and YPMDRL. Specifically, 10% of the negative blood samples found in the same prefecture were randomly selected for microscopic re-examination in prefecture-level laboratories. Then, 10% of the negative blood samples confirmed by prefecture-level laboratories were randomly selected for re-testing in YPMDRL only using microscopic examination mothed.

**Results：**

From 2016 to 2018, the planned number of people monitored in Yunnan Province should be 25 million, 303065 person times were actually monitored. 30307 negative blood samples should be re-tested in 16 prefecture-level laboratories, with 40021 samples actually re-tested and no false negative were found. 4002 negative blood samples confirmed in prefecture-level laboratories should be re-tested by YPMDRL, with 4801 samples actually re-tested and no false negative were found (Table 1).

| **Table 1. Re-testing of negative samples by YPMDRL in Yunnan Province (2016 to 2018)** | | | | | | | | | | | | | | |
| --- | --- | --- | --- | --- | --- | --- | --- | --- | --- | --- | --- | --- | --- | --- |
| **Areas** | **2016** | | | |  | **2017** | | | |  | **2018** | | | |
|  | **No. negative samples produced** | **Re-testing rate (%)** | **No. negative samples of ID** | **No.**  **negative samples of re-testing** |  | **No. negative samples produced** | **Re-testing rate (%)** | **No. negative samples of ID** | **No. negative samples of re-testing** |  | **No. negative samples produced** | **Re-testing rate (%)** | **No. negative samples of ID** | **No.**  **negative samples of re-testing** |
| DH | 66814 | 0.29 | 191 | 191 |  | 66746 | 0.26 | 174 | 174 |  | 50231 | 0.48 | 239 | 239 |
| BS | 22586 | 0.39 | 88 | 88 |  | 21153 | 0.43 | 91 | 91 |  | 22116 | 0.42 | 92 | 92 |
| KM | 16387 | 0.41 | 67 | 67 |  | 15284 | 0.62 | 94 | 94 |  | 15255 | 0.58 | 88 | 88 |
| LC | 40299 | 0.35 | 141 | 141 |  | 35648 | 0.40 | 144 | 144 |  | 29015 | 0.49 | 143 | 143 |
| PE | 25091 | 0.66 | 166 | 166 |  | 24030 | 0.57 | 137 | 137 |  | 24989 | 0.56 | 140 | 140 |
| DL | 8876 | 0.69 | 61 | 61 |  | 8285 | 0.74 | 61 | 61 |  | 8332 | 0.73 | 61 | 61 |
| QJ | 14241 | 0.70 | 99 | 99 |  | 13771 | 0.73 | 100 | 100 |  | 13271 | 0.75 | 100 | 100 |
| WS | 20621 | 0.61 | 126 | 126 |  | 19815 | 0.65 | 128 | 128 |  | 15989 | 0.80 | 128 | 128 |
| ZT | 18100 | 0.74 | 134 | 134 |  | 17100 | 0.76 | 130 | 130 |  | 17371 | 0.62 | 108 | 108 |
| XS | 12078 | 0.48 | 58 | 58 |  | 11628 | 0.47 | 55 | 55 |  | 13692 | 2.04 | 279 | 279 |
| LJ | 3600 | 0.72 | 26 | 26 |  | 3600 | 0.75 | 27 | 27 |  | 3600 | 0.89 | 32 | 32 |
| DQ | 3334 | 0.81 | 27 | 27 |  | 3452 | 0.70 | 24 | 24 |  | 3251 | 0.65 | 21 | 21 |
| NJ | 7196 | 0.76 | 55 | 55 |  | 8965 | 0.60 | 54 | 54 |  | 7198 | 0.76 | 55 | 55 |
| YX | 7819 | 0.92 | 72 | 72 |  | 7801 | 0.76 | 59 | 59 |  | 7735 | 0.63 | 49 | 49 |
| CX | 9479 | 0.60 | 57 | 57 |  | 8327 | 0.68 | 57 | 57 |  | 8273 | 0.68 | 56 | 56 |
| HH | 26544 | 0.64 | 170 | 170 |  | 25890 | 0.66 | 170 | 170 |  | 26920 | 0.62 | 167 | 167 |
| Total | 303065 | 0.51 | 1538 | 1538 |  | 291495 | 0.52 | 1505 | 1505 |  | 267238 | 0.66 | 1758 | 1758 |
| Note: (1) Areas: There are 16 prefectures (or city) , including Dehong(DH), Baoshan (BS), Kuming (KM), Lijiang (LJ), Pu’er (PE), Dali (DL), Xishuangbanna (XS), Nujiang (NJ), Weishan (WS), Honghe (HH), Zhaotong(ZT), Qujing (QJ), Licang (LC), Yuxi (YX), Chuxiong(CX), and Diqing(DQ); (2) ID: Initial Diagnosis; (3) YPMDRL: Yunnan Province Malaria Diagnosis Referent Laboratory. | | | | | | | | | | | | | | |
