# Supplementary material for: Analysis of initial laboratory diagnosis of malaria and its accuracy compared with re-testing from 2013 to 2018 in Yunnan Province, China
Source: Malar J. 2020 Nov 12;19:409. doi: 10.1186/s12936-020-03477-1 (PMC7664069; doi:10.1186/s12936-020-03477-1)
Supplement: Supplementary file 2 — Additional file 2. The details of nested PCR testing for differentiating between various Plasmodium species. [file 12936_2020_3477_MOESM2_ESM.docx]

| **Additional file 2 The details of nested PCR testing for differentiating between various *Plasmodium* species** | | | | | | |
| --- | --- | --- | --- | --- | --- | --- |
| Nested PCR | Specificity of primers | Primer name^△^ | Primer sequence^△^ | Expected PCR product (bp) | Reaction conditions | Reaction systems |
| First round | Genus | rPLU5 | 5’-CCTGTTGTTGCCTTAAACTTC-3； | 1200 | 94 ° C for 3min;94° C for 30s, 58° C for 30s, 72° C for 60s, 34 cycles; 72° C for 5min. | 25 ul reaction volume including 2.6 μl template, 14.0 μl 2 × PCR Mix hybrid system (Containing Taq enzyme), 0.7 μl upstream primer (20umol / L) and 0.7 μl downstream primers (20umol / L) |
|  |  | rPLU6 | 5’-TTAAAATTGTTGCAGTTAAAACG-3’ |  |  |  |
| Second round | *P. falciparum* | rFAL1 | 5’-TTAAACTGGTTTGGGAAAACCAAATATATT-3’ | 205 | 94 ° C for 3min;94° C for 30s, 60° C for 30s, 72° C for 60s, 34 cycles; 72° C for 5min |  |
|  |  | rFAL2 | 5’-ACACAATGAACTCAATCATGACTACCCGTC-3’ |  |  |  |
|  | *P. vivax* | rVIV1 | 5’-CGCTTCTAGCTTAATCCACATAACTGATAC-3’ | 120 |  |  |
|  |  | rVIV2 | 5’-ACTTCCAAGCCGAAGCAAAGAAAGTCCTTA-3’ |  |  |  |
|  | *P. malariae* | rMAL1 | 5’-ATAACATAGTTGTACGTTAAGAATAACCGC-3’ | 141 |  |  |
|  |  | rMAL2 | 5’-AAAATTCCCATGCATAAAAAATTATACAAA-3’ |  |  |  |
|  | *P. ovale* | rOVA1 | 5’-ATCTCTTTTGCTATTTTTTAGTATTGGAGA-3’ | 800 |  |  |
|  |  | rOVA2 | 5’-GGAAAGGACACATTAATTGTATCCTAGTG-3’ |  |  |  |
| ^△^: The primers’ name and sequence were cited from References [24]. | | | | | | |
