# Supplementary material for: Analysis of initial laboratory diagnosis of malaria and its accuracy compared with re-testing from 2013 to 2018 in Yunnan Province, China
Source: Malar J. 2020 Nov 12;19:409. doi: 10.1186/s12936-020-03477-1 (PMC7664069; doi:10.1186/s12936-020-03477-1)
Supplement: Supplementary file 3 — Additional file 3. Accuracy of initial malaria diagnoses in Yunnan Province prefectures (2013 to 2018). [file 12936_2020_3477_MOESM3_ESM.docx]

| **Additional file 3. Accuracy of initial malaria diagnoses in Yunnan Province prefectures (2013 to 2018)** | | | | | | | | | | | | | | | | | | | | |  |
| --- | --- | --- | --- | --- | --- | --- | --- | --- | --- | --- | --- | --- | --- | --- | --- | --- | --- | --- | --- | --- | --- |
| Areas. |  | 2013 | |  | 2014 | |  | 2015 | |  | 2016 | | |  | | 2017 | |  | 2018 | |  |
|  | No.  PTD | No.  PTD | No. TSA |  | No.  PTD | No.TSA |  | No.  PTD | No.TSA |  | No.  PTD | No.TSA |  | | No.  PTD | | No.TSA |  | No.  PTD | No.TSA | *χ2*, *P* |
|  |  |  | (TSAR) |  |  | (TSAR) |  |  | (TSAR) |  |  | (TSAR) |  |  |  |  | (TSAR) |  |  | (TSAR) |  |
| DH | 1375 | 257 | 246(95.7) |  | 233 | 221(94.8) |  | 284 | 278(97.8) |  | 232 | 227(97.8) | |  | | 231 | 224 (97.0) |  | 138 | 135(97.8) | 23.195, 0.0003 |
| BS | 732 | 176 | 167(94.9) |  | 183 | 175(95.6) |  | 204 | 194(95.1) |  | 89 | 86(96.6) | |  | | 46 | 42(91.3) |  | 34 | 30(88.2) | 5.019, 0.414 |
| KM | 170 | 28 | 19 (67.9) |  | 34 | 26 (76.5) |  | 31 | 20 (64.5) |  | 29 | 17 (58.6) | |  | | 29 | 20 (69) |  | 19 | 15 (78.9) | 3.522, 0.620 |
| LC | 120 | 34 | 33 (97.1) |  | 22 | 19 (86.4) |  | 23 | 20 (87.0) |  | 21 | 13 (61.9) | |  | | 12 | 11 (91.7) |  | 8 | 8 (100.0) | 15.815, 0.007 |
| PE | 65 | 27 | 25 (92.3) |  | 17 | 16 (94.1) |  | 8 | 7 (87.5) |  | 10 | 10(100.0) | |  | | 1 | 1(100.0) |  | 2 | 2(100.0) | 4.093, 0.536 |
| DL | 60 | 11 | 11(100.0) |  | 25 | 22(88.0) |  | 12 | 10 (83.3) |  | 6 | 5 (83.3) | |  | | 2 | 2 (100.0) |  | 4 | 3 (75.0) | 2.847, 0.724 |
| QJ | 20 | 5 | 3 (60.0) |  | 5 | 4 (80.0) |  | 6 | 4 (66.7) |  | 2 | 2 (100.0) | |  | | 1 | 1 (100.0) |  | 1 | 1 (100.0) | 2.223, 0.818 |
| WS | 32 | 8 | 4 (50.0) |  | 6 | 5 (83.3) |  | 4 | 3 (75.0) |  | 7 | 6 (85.7) | |  | | 5 | 4 (80.0) |  | 2 | 2 (100.0) | 4.052, 0.542 |
| ZT | 17 | 2 | 1 (50.0) |  | 1 | 1 (100.0) |  | 7 | 4 (44.4) |  | 2 | 1 (50.0) | |  | | 1 | 1 (100.0) |  | 4 | 3 (75.0) | 1.832, 0.872 |
| XS | 46 | 13 | 13 (100.0) |  | 6 | 5 (83.3) |  | 19 | 17 (89.5) |  | 4 | 4 (100.0) | |  | | 1 | 1 (100.0) |  | 3 | 2 (66.7) | 4.568, 0.471 |
| LJ | 21 | 7 | 7 (100.0) |  | 5 | 5 (100.0) |  | 6 | 6 (100.0) |  | 1 | 1 (100.0) | |  | | 2 | 2 (100.0) |  | - | - | - |
| DQ | 2 | - | - |  | 1 | 1 (100.0) |  | - | - |  | - | - | |  | | - | - |  | 1 | 1 (100.0) | - |
| NJ | 32 | 12 | 11 (91.7) |  | 11 | 9 (81.8) |  | 6 | 6 (100.0) |  | 2 | 2 (100.0) | |  | | - | - |  | 1 | 1 (100.0) | - |
| YX | 13 | 2 | 2 (100.0) |  | 3 | 3 (100.0) |  | 2 | 0 |  | 2 | 2 (100.0) | |  | | 2 | 1 (100.0) |  | 2 | 0 | - |
| CX | 13 | 1 | 1 (100.0) |  | 2 | 2 (100.0) |  | 5 | 3 (60.0) |  | 3 | 3 (100.0) | |  | | - | - |  | 2 | 2 (100.0) | - |
| HH | 23 | 4 | 3 (75.0) |  | 2 | 2 (100.0) |  | 2 | 2 (100.0) |  | 4 | 4 (100.0) | |  | | 6 | 3 (50.0) |  | 5 | 5 (100.0) | 7.339, 0.197 |
| Total | 2742 | 588 | 547 (93.0) |  | 556 | 516(92.8) |  | 619 | 574 (92.7) |  | 414 | 383 (92.5) | |  | | 339 | 313 (92.3) |  | 226 | 210 (92.9) | 0.203, 0.999 |
| Note: (1) Areas: There are 16 prefectures (or city) including DH prefecture, BS city, KM city, LC city, PE city, DL prefecture, QJ city, WS prefecture, ZT city, XS prefecture, LJ city, DQ prefecture, NJ prefecture, YX city, CX prefecture and HH prefecture. (2) ID: Initial diagnosis, undertaken by county laboratory; PTD: Parasitological re-detection diagnosis, undertaken by province referent laboratory; TSA: Test species accuracy comparison ID with PTD for assessment the accuracy of the initial diagnosis; TSAR: The rate of TSA. | | | | | | | | | | | | | | | | | | | | | |
