# Supplementary material for: Analysis of initial laboratory diagnosis of malaria and its accuracy compared with re-testing from 2013 to 2018 in Yunnan Province, China
Source: Malar J. 2020 Nov 12;19:409. doi: 10.1186/s12936-020-03477-1 (PMC7664069; doi:10.1186/s12936-020-03477-1)
Supplement: Supplementary file 4 — Additional file 4. Analysis of the inconsistency between initial diagnosis and re-testing in Yunnan Province from 2013 to 2018. [file 12936_2020_3477_MOESM4_ESM.docx]

| **Additional file 4. Analysis of the inconsistency between initial diagnosis and re-testing in Yunnan Province from 2013 to 2018** | | | | | | | | | | | | |
| --- | --- | --- | --- | --- | --- | --- | --- | --- | --- | --- | --- | --- |
| Years | No.PTD | No. NA  (NAR) | Re-testing results for incorrect *P.f* | | | |  | Re-testing results for incorrect *P.v* | | | | |
|  |  |  | No.NA | Neg  (CR) | *P.v*  (CR) | *P.o*  (CR) |  | No.NA | Neg  (CR) | *P.f*  (CR) | *P.o*  (CR) | *P.m*  (CR) |
| 2013 | 588 | 41 (6.9) | 16 | 5 (31.3) | 11 (68.7) | 0 |  | 25 | 13 (52.0) | 9 (36.0) | 1 (4.0) | 2 (8.0) |
| 2014 | 556 | 40 (7.2) | 13 | 11 (84.6) | 1 (7.7) | 1 (7.7) |  | 27 | 15 (55.6) | 8 (29.6) | 0 | 4 (14.8) |
| 2015 | 619 | 45 (7.3) | 21 | 9 (42.9) | 12 (57.1) | 0 |  | 24 | 11 (45.8) | 10 (41.7) | 2 (8.3) | 1 (4.2) |
| 2016 | 414 | 31 (7.5) | 13 | 8 (61.5) | 5 (38.5) | 0 |  | 18 | 7 (38.9) | 10 (55.6) | 1 (5.5) | 0 |
| 2017 | 339 | 26 (7.7) | 7 | 6 (85.7) | 1 (14.3) | 0 |  | 19 | 9 (47.4) | 7 (36.8) | 2 (10.5) | 1 (5.3) |
| 2018 | 226 | 16 (7.1) | 5 | 5 (100.0) | 0 | 0 |  | 11 | 4 (36.4) | 1 (9.0) | 4 (36.4) | 2 (18.2) |
| Total | 2742 | 199 (7.3) | 75 | 44 (58.7) | 30 (40.0) | 1 (1.3) |  | 124 | 59 (47.6) | 45 (36.2) | 10 (8.1) | 10 (8.1) |
| *χ2*, *P* |  | 0.191,  0.999 |  |  |  |  |  |  |  |  |  |  |
| Note: ID: Initial diagnosis, undertaken by county laboratory; NA: Specie incorrect of initial diagnosis comparison with parasitological re-testing diagnosis by YPMDRL; NAR: The rate of NA. Neg: test negative by parasitological re-testing diagnosis; *P.f*, *P.v*, *P.o* and *P.m*: *P.falciparum*, *P. vivax*, *P.ovale* and *P. malariae*, respectively.CR: Constituent ratio. | | | | | | | | | | | | |
